# Granulomatous Hepatitis Secondary to Histoplasmosis in an Immunocompetent Patient

**DOI:** 10.7759/cureus.17631

**Published:** 2021-09-01

**Authors:** Adel Muhanna, Faisal M Nimri, Zaid A Almomani, Laith Al Momani, Alisa Likhitsup

**Affiliations:** 1 Internal Medicine, University of Missouri Kansas City, Kansas City, USA; 2 Internal Medicine, Henry Ford Health System, Detroit, USA; 3 Internal Medicine, Jordan University of Science and Technology, Irbid, JOR; 4 Gastroenterology, University of Missouri Kansas City, Kansas City, USA; 5 Gastroenterology and Hepatology, University of Missouri Kansas City, Kansas City, USA

**Keywords:** endemic mycosis, granulomatous hepatitis, gastrointestinal histoplasmosis, elevated liver enzyme, immuno-competent

## Abstract

*Histoplasma capsulatum* is the most common endemic mycosis in the United States and usually occurs in certain geographic areas, such as the Mississippi or Ohio River valleys. Histoplasmosis usually causes a mild disease in the immunocompetent but can progress to disseminated disease in patients with impaired immunity. Granulomatous hepatitis as a manifestation of disseminated histoplasmosis in immunocompetent patients is extremely rare. We report the case of a 62-year-old immunocompetent gentleman with a history of histoplasmosis who presented with abdominal pain, elevated liver enzymes, who was diagnosed with granulomatous hepatitis secondary to histoplasmosis.

## Introduction

Histoplasmosis, caused by *Histoplasma capsulatum* var. capsulatum, is currently the most common endemic mycosis in the United States (US), with an estimated incidence of 500,000 cases annually in the US [1]. Although histoplasmosis occurs primarily in the Midwestern and Central areas of the US, especially along the Mississippi and Ohio River Valleys, these infections can be found in other areas across the country [2,3]. While most patients infected with histoplasmosis have asymptomatic or mild disease, around 5% of patients develop symptomatic pulmonary infections that can progress to severe disseminated infection in patients with compromised immunity [4]. Gastrointestinal manifestations are found in less than 10% of patients, with liver involvement being even less common [5]. Herein, we present the case of a 62-year-old immunocompetent man who presented with transaminitis secondary to histoplasmosis. 

## Case presentation

**Figure 1:**
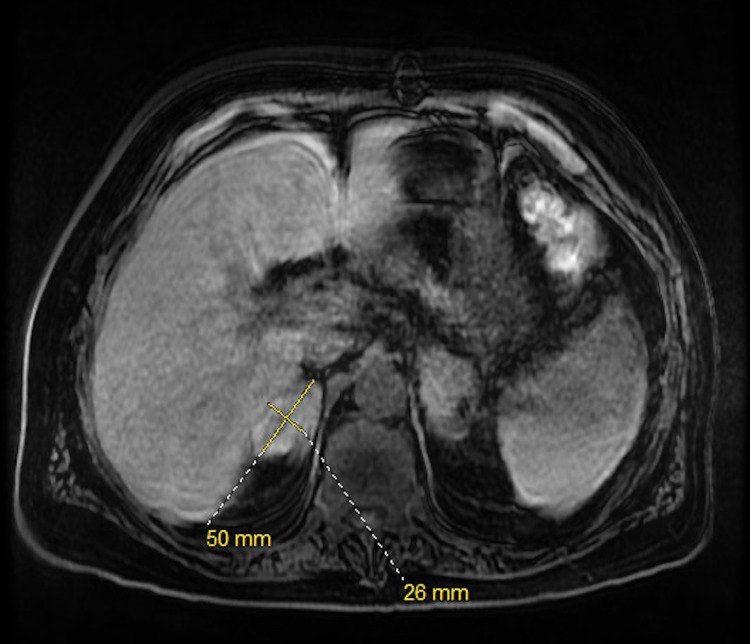
Transversal T1-weighted magnetic resonance image of the abdomen after contrast Transversal T1-weighted magnetic resonance image of the abdomen after contrast showing right adrenal mass measuring 5 cm x 2.6 cm

**Figure 2:**
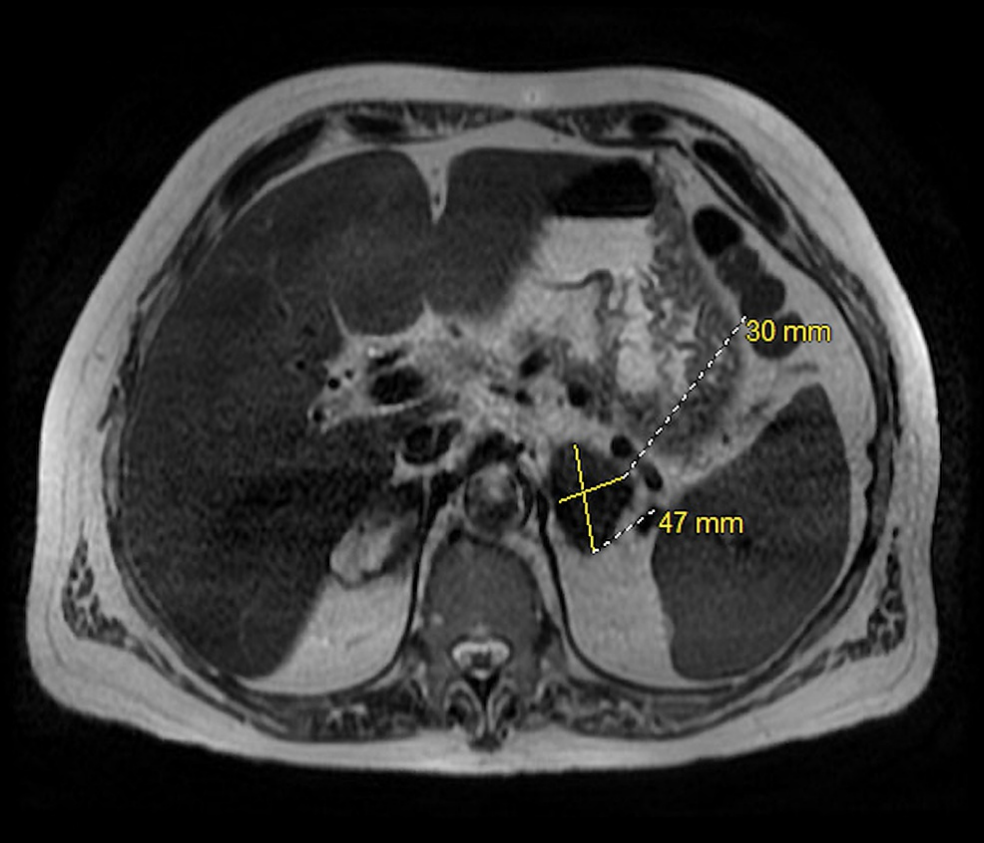
Transversal T1-weighted image of the abdomen without contrast Transversal T1-weighted image of the abdomen without contrast showing left adrenal mass measuring 4.7 cm x 3.0 cm

**Figure 3:**
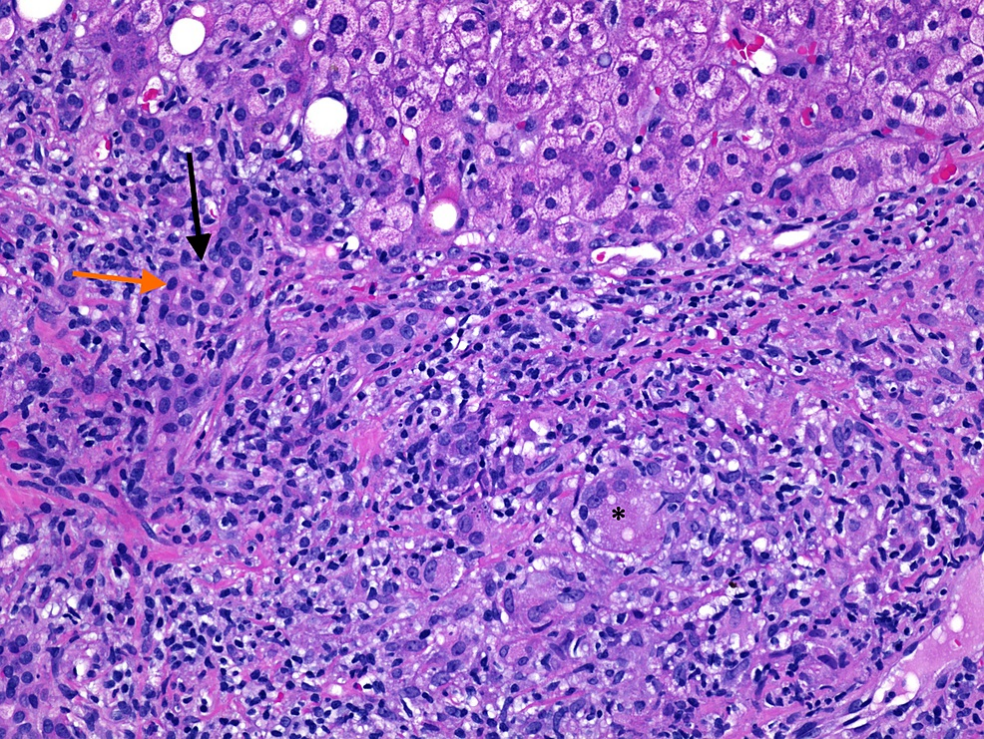
Hematoxylin and Eosin (H&E) stain of liver biopsy (H&E stain 20x). Marked bile duct reaction (black arrow) with reactive changes, foci of lymphocytic cholangitis (orange arrow), and portal granuloma with giant cells (asterisk).

**Figure 4:**
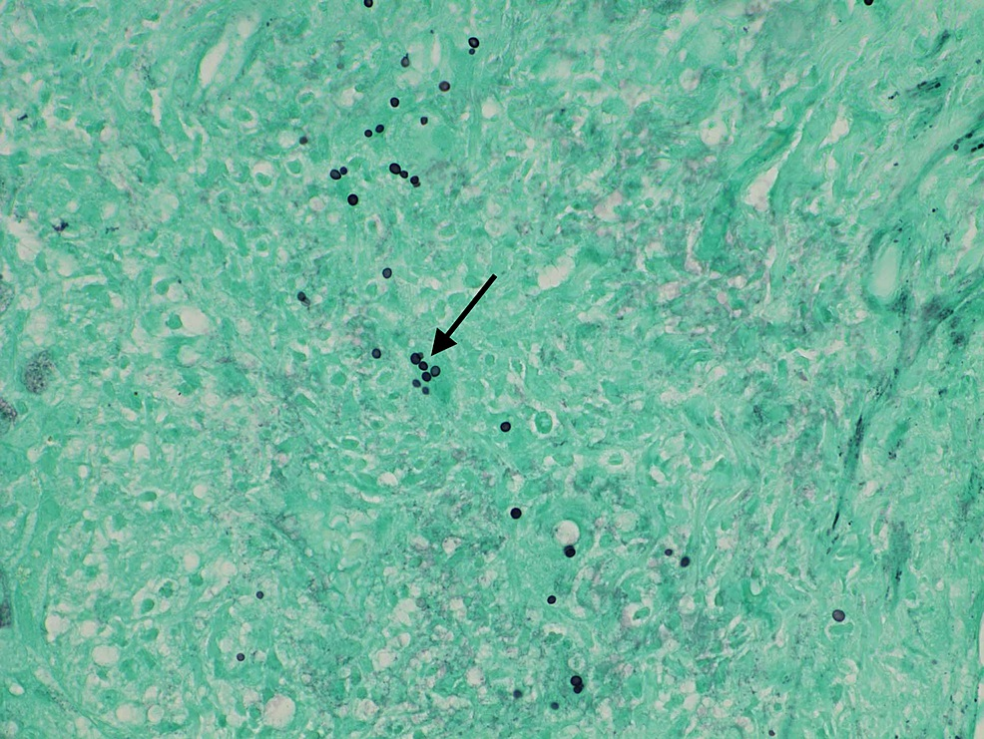
Grocott’s methenamine silver (GMS) stain of liver biopsy (GMS stain 40x). Scattered fungal yeast forms presented within portal areas in association with portal-based granulomas, morphologically consistent with *Histoplasma* species.

A 62-year-old gentleman with significant past medical history of gastroesophageal reflux disease who presented to the emergency department with right upper quadrant abdominal pain, associated with nausea, low appetite and loose stool for one month prior to his presentation. He denies any fever or chills. His medications include proton pump inhibitors for his acid reflux otherwise denied history of new medication. He is a retired mailman who did not have any house pet. There was no recent travel history or sick exposure. The patient is a current cigarette smoker, and he denies alcohol consumption or illicit drug use. Physical exam revealed afebrile, mild sinus tachycardia, a well-appearing alert and oriented male without jaundice yet had prominent signs of chronic pruritus. Laboratory tests revealed unremarkable basic metabolic panel, aspartate transaminase (AST) of 321 IU/L, alanine aminotransferase (ALT) of 228 IU/L, alkaline phosphate (ALP) 462 IU/L, total bilirubin of 2.1 mg/dl, direct bilirubin 0.2 mg/dL, white blood cells (WBC) of 4.30 K/ml, platelets of 148 K/ml, and an international normalized ratio (INR) of 1.1. The patient's work was negative for acute and chronic viral hepatitis, human immunodeficiency virus, cytomegalovirus. Chronic liver disease markers including antinuclear antibodies, anti-smooth muscle antibodies, anti-mitochondrial antibodies, ceruloplasmin, alpha-1 antitrypsin, and iron studies were unremarkable. Magnetic Resonance Imaging (MRI) of the abdomen showed a normal liver, bilateral adrenal masses (Figures 1, 2), splenomegaly and incidental moderate gallbladder wall thickening without gallstones. Ultrasonography (USG) of the abdomen was suboptimal and decision was made to get Endoscopic Ultrasonography (EUS) which showed findings of gallbladder sludge and fatty liver. Cholecystectomy was recommended to address patient symptoms and gallbladder sludge with concurrent liver biopsy to evaluate elevated liver function tests (LFTs). Of note, Computed Tomography (CT) scan of the chest was done and it showed no lung lesions. The patient underwent laparoscopic cholecystectomy with liver biopsy. The pathology showed chronic cholecystitis and liver biopsy showed marked bile duct reaction with reactive changes consisting of portal tract inflammatory cell infiltration, with a foci of lymphocytic cholangitis as well as a few lymphocytes in sinusoids, portal granuloma with giant cells, and fibrotic changes in the liver (Figure 3). Grocott’s methenamine silver (GMS) stain showed scattered fungal yeast forms within portal areas in association with portal-based granulomas, morphologically consistent with *Histoplasma* species (Figure 4). Acid-fast bacilli staining was negative for acid-fast organisms. These histologic findings were consistent with granulomatous hepatitis secondary to histoplasmosis. The patient was started on daily treatment of itraconazole followed by regular follow-ups. The patient's right upper quadrant abdominal pain has resolved after cholecystectomy and his laboratory follow-up results have normalized. His most recent LFTs were obtained 9 months from starting itraconazole and showed an ALT of 16 IU/L, AST of 24 IU/L, ALP of 101 IU/L, total bilirubin of 1.2 mg/dl, and direct bilirubin of 0 mg/dl.

## Discussion

*Histoplasma capsulatum* is a thermally dimorphic fungus, found as a mold in the environment and as a yeast at 37ºC [6]. Infection develops when the spores are inhaled into the lungs. Histoplasmosis is classified into pulmonary and extrapulmonary or disseminated histoplasmosis (DH). Cell-mediated immunity plays the most important role in recovery from histoplasmosis, either by killing the organism or by forming a calcified granuloma [7], and failure to achieve cell-mediated immunity is one of the key defects in patients with DH. Unlike in our case, DH is mostly found in immunocompromised patients, such as those with AIDS, primary immunodeficiencies, and solid organ transplant recipients [8]. 

The presentation of histoplasmosis in symptomatic patients most commonly resembles community-acquired pneumonia and usually consists of mild to moderate symptoms of cough, fever, dyspnea, headache, and arthralgia. However, the clinical presentation of DH can differ between individuals, and patients with acute infection can present with fatigue, fever, pancytopenia or coagulopathy, and hepatosplenomaegaly [3]. Adrenal involvement occurs in approximately 80% of patients with DH but adrenal insufficiency is found in the minority of cases [9], which may explain our patient's splenomegaly bilateral adrenal assess on CT imaging. Even though gastrointestinal involvement is common in DH, gastrointestinal disease is rarely identified and causes clinical symptoms only in 3 to 12% of patients [10]. The most common gastrointestinal findings include mucosal ulcerations that can be located anywhere from the mouth to the anus, colonic perforation, and masses that may resemble malignancies [11]. Liver involvement in histoplasmosis is even less commonly reported. Granulomatous hepatitis has a wide range of differential diagnoses including autoimmune, drug-related, infectious, a manifestation of systematic diseases, and idiopathic [12]. Therefore, the diagnosis and management can be challenging, and physicians must have a high index of suspicion because of the high fatality in patients with DH if left untreated.

To our knowledge, there are very few cases in the literature describing granulomatous hepatitis due to histoplasmosis, especially in immunocompetent adults [13- 18]. One case reported a previously healthy man who presented with fever, hepatomegaly, and anorexia and was found to have granulomatous hepatitis due to acute histoplasmosis [13]. Another case was that of a 66-year-old female who presented with fever of unknown origin and an elevation in her liver enzymes as a primary presentation of histoplasmosis. However, unlike our case, the patient had a past medical history of cell-mediated immunodeficiency, a known risk factor for DH [14]. The diagnosis in both cases was established by tissue sampling. Despite the majority of literature reporting cases of granulomatous hepatitis in immunocompromised patients [15- 18], the workup of our patient was negative to tests that would indicate immunodeficiency, such as HIV, autoimmune, and chronic medical conditions. 

Treatment is indicated for all patients with disseminated histoplasmosis. The management depends on disease severity and organ involvement and is typically with anti-fungal agents. Itraconazole should be administered three times daily for three days, and then daily maintenance treatment that is optimally continued for a year [19]. Amphotericin B can be used when there is severe disease with central nervous system involvement [20]. Fluconazole has not been found effective in the case of DH as itraconazole and is not used routinely for treatment [21]. Patients who received fluconazole developed resistance faster than with other antifungal agents [22]. In our case, the patient was continued on daily itraconazole as well as regular follow-ups, and his LFTs normalized nine months after treatment with itraconazole.

## Conclusions

The diagnosis of liver involvement in histoplasmosis can be challenging, which may lead to inappropriate therapy or unnecessary interventions and liver biopsy may be considered in the presence of elevated liver function tests if the diagnosis was not achieved by less invasive testing as clinically indicated. Treatment with systematic antifungal medications is highly effective for these patients.
